# Proton pump inhibitors and risk of gastric cancer: a population-based cohort study

**DOI:** 10.1038/sj.bjc.6605024

**Published:** 2009-04-07

**Authors:** A H Poulsen, S Christensen, J K McLaughlin, R W Thomsen, H T Sørensen, J H Olsen, S Friis

**Affiliations:** 1 Institute of Cancer Epidemiology, Danish Cancer Society, Strandboulevarden 49, DK-2100 Copenhagen, Denmark; 2 Department of Clinical Epidemiology, Aarhus University Hospital, Ole Worms allé 150, DK-8000 Aarhus, Denmark; 3 International Epidemiology Institute, 1455 Research Boulevard, Rockville MD 20850, USA; 4 Department of Medicine, Vanderbilt University Medical Center, Vanderbilt-Ingram Comprehensive Cancer Center, Nashville, TN 37232, USA

**Keywords:** stomach neoplasms, proton pump inhibitors, histamine-2-antagonists, risk, cohort study

## Abstract

Proton pump inhibitor (PPI) use leads to hypergastrinaemia, which has been associated with gastrointestinal neoplasia. We evaluated the association between PPI use and risk of gastric cancer using population-based health-care registers in North Jutland, Denmark, during 1990–2003. We compared incidence rates among new users of PPI (*n*=18 790) or histamine-2-antagonists (H2RAs) (*n*=17 478) and non-users of either drug. Poisson regression analysis was used to estimate incidence rate ratios (IRRs) adjusted for multiple confounders. We incorporated a 1-year lag time to address potential reverse causation. We identified 109 gastric cancer cases among PPI users and 52 cases among H2RA users. After incorporating the 1-year lag time, we observed IRRs for gastric cancer of 1.2 (95% CI: 0.8–2.0) among PPI users and 1.2 (95% CI: 0.8–1.8) among H2RA users compared with non-users. These estimates are in contrast to significant overall IRRs of 9.0 and 2.8, respectively, without the lag time. In lag time analyses, increased IRRs were observed among PPI users with the largest number of prescriptions or the longest follow-up compared with H2RA users or non-users. Although our results point to a major influence of reverse causation and confounding by indication on the association between PPI use and gastric cancer incidence, the finding of increased incidence among PPI users with most prescriptions and longest follow-up warrants further investigation.

There has been some concern about the long-term safety of proton pump inhibitors (PPIs). The profound reduction in gastric acid secretion induced by PPIs leads to increased secretion of gastrin, and most PPI users have moderate hypergastrinaemia (Lamberts *et al*, 1988; Klinkenberg-Knol *et al*, 1994). Gastrin has trophic effects on the gastrointestinal mucosa, and hypergastrinaemia has been associated with an increased risk of gastric carcinoids, and gastric and colonic carcinomas (Havu, 1986; Laine *et al*, 2000; Gillen and McColl, 2001; Waldum *et al*, 2005; Kuipers, 2006), although recent results for PPI use and colorectal cancer risk are reassuring (Robertson *et al*, 2007; Yang *et al*, 2007; van Soest *et al*, 2008). Hyperplasia of enterochromaffin-like cells has been seen in long-term PPI users (Lamberts *et al*, 1993; Eissele *et al*, 1997; Klinkenberg-Knol *et al*, 2000); and in patients with *Helicobacter pylori* (*H. Pylori*) infection, long-term PPI use has been associated with an increased incidence of atrophic gastritis (Kuipers *et al*, 1996), a precursor of gastric adenocarcinoma (Uemura *et al*, 2001; Ye and Nyren, 2003).

Few epidemiological studies have evaluated the association between PPI use and gastric cancer. Two studies based on UK populations reported an increased risk of gastric cancer among users of PPI that was attributed to probable reverse causation or confounding by indication (Bateman *et al*, 2003; Garcia Rodriguez *et al*, 2006). Given the widespread use of PPI and the unresolved questions about their carcinogenic potential, we examined the association between PPI use and the risk of gastric cancer in a large population-based cohort of Danish PPI users.

## Materials and methods

We conducted this population-based cohort study within North Jutland County, Denmark (∼500 000 inhabitants) in 1990–2003. The Danish National Health-care System provides all Danish citizens with unrestricted access to tax-supported public medical services and reimbursement of most prescribed drugs, including PPIs and histamine-2-antagonists (H2RAs). All medical services are registered to individual patients by use of a civil registry number assigned to all Danish citizens, which encodes gender and date of birth. Its use allows identification of complete prescription and hospitalisation histories for each individual.

Using the Danish Civil Registration System that has provided information on vital status and residence for the entire population since 1968, we identified all individuals aged 40–84 years during the study period and resident in North Jutland County on 1 January 1989. We excluded all individuals with a history of cancer (except non-melanoma skin cancer) before study entry (1990 or age 40) by linkage to the Danish Cancer Registry, which provides accurate and virtually complete nationwide ascertainment of cancers (Storm *et al*, 1997). In an attempt to include only new users of acid-suppressive drugs (Ray, 2003), all individuals who filed prescriptions for PPI or H2RA during 1989 or before turning 40 during the study period (i.e., before becoming eligible for analysis) were excluded from the study population. The final study population comprised of 280 872 individuals.

In North Jutland County, prescription data from all pharmacies are electronically transferred to a research database at Aarhus University Hospital, initiated in 1989 (Gaist *et al*, 1997). The database holds key information on all prescriptions dispensed for reimbursable drugs, including the type and amount of drug (according to the ATC classification system), date of dispensing at the pharmacy and the individual's civil registry number. Use of PPI (ATC code A02BC) was defined as filing ⩾2 PPI prescriptions during the study period and H2RA use similarly (ATC code A02BA). Non-use of drug was defined as less than two prescriptions for either drug; mixed use (⩾2 prescriptions of both PPI and H2RA) was not evaluated. We were not able to obtain valid information on indication for use of PPI and H2RA as indication for use was not recorded in the prescription database.

From the database, we identified patients who had undergone *H. Pylori* eradication therapy, namely those who within the study period filed simultaneous prescriptions for any two of the three antibiotics (amoxicillin, clarithromycin and metronidazole; ATC codes: J01CA04, J01FA09 and P01AB01) used in triple therapy (Malfertheiner *et al*, 2006). In addition, we obtained prescription data for non-steroidal anti-inflammatory drugs (NSAIDs) (ATC codes: M01A, N02BA01, N02BA51 and B01AC06).

All patients who had undergone gastroscopy (Danish Classification of Surgical Procedures and Therapies (Danish Board of Health, 1989, 1995) codes: 91010 (1977–1995); UJD02, UJD05 (1996–2005)) in North Jutland County were identified from the County Hospital Discharge Registry containing information on all non-psychiatric hospital admissions (since 1977) and out-patient visits (since 1995) (Andersen *et al*, 1999). If the exact date of gastroscopy was not available, we used the first day of admission. To exclude registrations of gastroscopy used in the diagnosis of gastric cancer cases, we excluded examinations performed ⩽1 year preceding censoring event, by adding 1 year to all gastroscopy dates.

Proxy measures of smoking and alcohol abuse were assessed from information in the hospital and prescription registers. For heavy smoking individuals, we obtained information on first admission for chronic obstructive pulmonary disorder (COPD) (ICD-8: 490–492; ICD-10: J40–44). For heavy alcohol use individuals, we identified the date of first recorded prescription for drugs used to treat alcohol addiction (disulfiram, acamprosate and naltrexone) (ATC codes: N07BB01, 03, 04; V03AA01, 02) or admission for alcohol-related diseases, including psychiatric and neurological conditions, gastrointestinal disease and alcohol poisoning (ICD-8: 291, 303, 57109, 57110, 57710, 979, 980; ICD-10: F10, G312, G621, G721, I426, K292, K70, K860, R780, T51, Z721).

Information on first primary diagnoses of gastric and other cancers was obtained from the Danish Cancer Registry. Gastric cancer cases were identified by the topographical head category 151 according to a modified Danish version of the International Classification of Diseases, seventh revision (ICD-7). We were not able to meaningfully evaluate associations by gastric subsite, as more than 40% of cancer cases were classified as unspecified gastric cancers (ICD-7: 151.0).

Subjects entered the study population on 1 January 1990 or age 40, whichever occurred latest, and were censored on first occurring date of primary gastric cancer, other primary cancer (except non-melanoma skin cancer), age 85, death, migration from North Jutland County, 31 December 2003 or, for PPI or H2RA users, at second prescription for the other agent (i.e., mixed use).

The person time of the study subjects was broken down according to PPI or H2RA use in exposed time (⩾2 prescriptions) (PPI or H2RA use) and unexposed time (<2 prescriptions) (non-use of both PPI and H2RA). Person time between first and second PPI or H2RA prescription was treated as unexposed. Person time for PPI or H2RA use was further categorized into four exposure periods based on duration of follow-up since the second prescription (<1, 1, 2–4 and 5+ years). Person time for PPI or H2RA use was also categorized according to the number of prescriptions filled (2–4, 5–14 and 15+ prescriptions).

### Statistical analysis

Log-linear Poisson regression analysis was used to compute incidence rate ratios (IRRs) for gastric cancer among PPI and H2RA users compared with non-users. To minimise the potential influence of protopathic bias (reverse causation) on PPI use and gastric cancer, we incorporated a 1-year lag time in the main analyses, by subtracting 1 year from the date of gastric cancer diagnosis or censoring dates, except for age and shift of treatment. In an attempt to account for unmeasured confounding factors, we also computed IRR estimates in direct comparisons of PPI and H2RA users in equivalent strata of prescription frequency and length of follow up.

All analyses were adjusted for calendar period (1990–1996, 1997–2003), gender, age (40–49, 50–59, 60–69 and 70–85 years), history of *H. pylori* eradication therapy (yes/no), gastroscopy (⩾1 year before gastric cancer diagnosis or other censoring event) (yes/no), COPD (yes/no), alcohol-related admission or therapy (yes/no) and ever use of NSAIDs (0–1, 2+ prescriptions). The proxy variables for heavy smoking and alcohol abuse had no appreciable effect on the risk estimates and were therefore dropped from the final models. Subjects were allowed to change between categories of covariates and exposure variables over time. Within each categorical level all variables were treated as time independent.

The statistical analyses were performed in SAS 9.1.

## Results

We identified 18 790 new users of PPI with less than two earlier recorded prescriptions for H2RA and 17 478 new users of H2RA with less than two earlier recorded PPI prescriptions. After incorporation of the 1-year lag time, 15 065 new PPI users and 16 176 new H2RA users remained. Characteristics of the groups are presented in Table 1. PPI users were slightly older than H2RA users and had slightly higher use of NSAIDs. Of PPI users, 13 and 4% of H2RA users, had undergone *H. pylori* eradication therapy. A record of gastroscopy (⩾1 year before censoring events) was found among 47% of PPI users, 33% of H2RA users (Table 1) and 11% of the total study population (results not shown). Use of PPI increased markedly during the study period. Omeprazole accounted for the majority of PPI use, whereas cimetidine was the most frequently prescribed H2RA. A similar distribution of characteristics was found in the lagged study population (data not shown).

Overall, PPI users accrued 66 630 person-years with a mean follow-up of 3.5 years (range: 0–13.8 years), and H2RA users accrued 90 904 person-years (mean: 5.2, range: 0–13.9 years). After incorporating the 1-year lag time, the corresponding person-years were 51 854 (mean: 3.4, range: 0–12.8 years) among PPI users and 81 256 (mean: 5.0, range: 0–12.9 years) among H2RA users. In the lag time analysis, 1111 PPI users and 5673 H2RA users were censored because of use of the other agent during follow up. We observed 109 cases of gastric cancer among PPI users and 52 cases among H2RA users. The overall (not taking lag time into account) IRR of gastric cancer was 9.0 (95% CI: 6.9–11.7) among PPI users and 2.8 (95% CI: 2.0–3.7) among H2RA users, compared with non-users (<2 prescriptions) of both PPI and H2RA (data not shown).

Results of the lag time analysis are presented in Table 2. The IRR of gastric cancer was 1.2 (95% CI: 0.8–2.0; based on 24 exposed cases) among PPI users and 1.2 (95% CI: 0.8–1.8; 30 exposed cases) among H2RA users, compared with non-users. Comparison between PPI users and H2RA users yielded an overall IRR for PPI use of 1.3 (95% CI: 0.7–2.3) Stratifying PPI users by duration of follow-up yielded increased IRRs for gastric cancer with less than 1 year of follow-up compared with non-users (IRR 2.3, 95% CI: 1.2–4.3) or H2RA users (2.4 95% CI: 0.7–8.0). In intermediate follow-up periods (1–4 years), the corresponding IRRs were below unity in both comparisons, whereas PPI users with 5 or more years of follow-up experienced an IRR of 2.3 (95% CI: 1.2–4.3) when compared with non-users and 1.8 (95% CI: 0.6–5.0) when compared with H2RA users.

Stratification showed increasing IRRs with increasing number of prescriptions when compared with non-use of acid-suppressing drugs, from 0.8 (95% CI: 0.4–1.6) with 2–4 PPI prescriptions to 2.1 (95% CI: 1.0–4.7) with 15 or more prescriptions. When compared with H2RA users with equivalent number of prescriptions, however, no clear pattern emerged and the IRR associated with 15 or more prescriptions was 1.4 (95% CI: 0.5–4.3).

Stratification of PPI users in the lag time analysis by history/no history of *H. pylori* eradication yielded IRRs of 3.3 (*n*=7; 95% CI: 1.5–7.2) and 1.1 (*n*=17; 95% CI: 0.7–1.9), respectively, when compared with non-users of acid-suppressing drugs (data not shown).

The vast majority of the gastric cancers were adenocarcinomas. Two gastric tumours observed in PPI users were classified as carcinoids and both were diagnosed within 1 year of the second PPI-prescription. No carcinoids were observed among H2RA users.

## Discussion

In this prospective, population-based cohort study risk estimates for gastric cancer among users of either PPI or H2RA were close to unity after incorporating a 1-year lag time. This is in contrast to a substantially increased overall incidence of gastric cancer among users of either PPI or H2RA in analysis without lag time. In the lagged analyses, we observed increased risk estimates among PPI users with most prescriptions or longest follow-up when compared with non-users of acid-suppressive drugs or with H2RA users.

Earlier studies of acid-suppressive drugs and gastric cancer have also reported an increased risk of gastric cancer associated with PPI use beyond the first year of treatment (Bateman *et al*, 2003; Garcia Rodriguez *et al*, 2006). In a cohort of 18 000 British PPI users, a sevenfold increased risk of death from gastric cancer was found, which disappeared by the fourth year of follow-up, indicating that this was because of confounding by indication rather than a causal relationship with PPI use (Bateman *et al*, 2003). A recent case–control study, based on the General Practice Research Database, reported that current PPI users with more than 3 years of use experienced a threefold increased risk (OR 3.0, 95% CI: 1.0–9.0) of non-cardia gastric cancer, whereas current long-term users of H2RA had no excess risk (OR 0.9, 95% CI: 0.5–1.8) (Garcia Rodriguez *et al*, 2006). The excess risk among PPI users was largely restricted to patients with ulcer indications. The authors argued that as gastric ulcers are associated with gastric neoplasia it was not possible to determine if the excess cancer risk was attributable to the underlying ulcer or to PPI use (Garcia Rodriguez *et al*, 2006; McColl, 2006).

Our finding of excess gastric cancer among with the greatest number of PPI prescriptions or longest follow-up, even after taking a 1-year lag time into account, would be compatible with a causal association with gastric cancer. However, a more likely explanation is confounding by indication, with *H. pylori* infection acting as the underlying risk factor associated with both gastric ulcer – and thus PPI treatment – and gastric cancer (13% of PPI users *vs* 4% H2RA users had undergone eradication therapy). Our observation that the excess risk among PPI users was largely confined to individuals with an earlier history of *H. pylori* eradication (IRR=3.3) supports this interpretation. On the other hand, increased risk estimates for gastric cancer with extended follow-up were also seen when compared with H2RA users who are likely to be more similar to PPI users in terms of underlying indications and comorbid conditions, although these estimates were based on small numbers. Moreover, as PPIs are more potent than H2RAs, there may be potential for confounding by severity of disease in the comparison of PPI and H2RA use.

Our study had the advantage of collecting information from population-based databases with virtually complete data on drug prescriptions and cancer diagnoses, thus minimising the possibility of selection and information biases. Another strength was our ability to apply a new-user design (Ray, 2003) with limited loss of eligible PPI users, as marketing of PPIs first began in 1989 and PPIs were only available on prescription during the study period. H2RA was available over-the-counter throughout the study period, however, individuals obtaining H2RA by prescription presumably included most long-term users and likely had an indication pattern similar to that of PPI users. Finally, our exposure definition of two or more prescriptions for either PPI or H2RA makes non-compliance unlikely.

The main limitations were the small number of long-term users of PPI, our inability to address subtypes of gastric cancer, and our inability to adjust for indication of use of PPI and H2RA. We were able to evaluate the influence of *H. pylori* infection, but only for those study subjects who underwent eradication therapy, thus residual confounding by untreated *H. pylori* infection may have influenced our results. We adjusted the risk estimates for NSAID use, which has been associated with a reduced risk of gastric cancer (Wang *et al*, 2003), but we were not able to adjust for dietary factors, and only proxy measures for tobacco and alcohol use. Finally, our study had relatively low-statistical precision.

The increased incidence of gastric cancer associated with PPI use observed in this and earlier studies is likely to result from confounding by indication; nevertheless, we cannot rule out the possibility of a causal association between long-term PPI use and risk of gastric cancer. Larger studies of long-term PPI use would be required to clarify this issue.

## Figures and Tables

**Table 1:** Characteristics of exclusive users of PPIs and H2RAs

	**PPI**		**H2RA**	
	***n***	**%**	***n***	**%**
*Total*				
Persons	18 790	100	17 478	100
Females	10 026	53	9463	54
Males	8764	47	8015	46
				
*Age*				
40–49	3805	20	4052	23
50–59	4663	25	4262	24
60–69	4328	23	4130	24
70+	5994	32	5034	29
Mean	62		61	
				
*Calendar year*				
1990–1991	353	2	3603	21
1992–1993	1253	7	4927	28
1994–1995	2141	11	3538	20
1996–1997	3001	16	2220	13
1998–1999	3597	19	1510	9
2000–2001	3834	20	944	5
2002–2003	4611	25	736	4
				
*Number of prescriptions*				
2–4	9155	49	7970	46
5–14	5624	30	5152	29
15+	4011	21	4356	25
				
*Other medical treatment or procedures*				
NSAID use	14 403	77	11 761	67
*H. pylori* eradication^a^	2371	13	694	4
Gastroscopy^b^	8861	47	5768	33
				
*Lifestyle factors* c				
COPD	1034	6	865	5
Alcoholism	1099	6	914	5
				
*Types of H2RA used* d				
Cimetidine			13 826	79
Ranitidine			1684	10
Nizatidine			1029	6
Famotidine			15	0
Ranitidine bismuth citrate			5	0
Mixed use			919	5
				
*Types of PPI used* d				
Omeprazole	9263	49		
Lanzoprazole	3366	18		
Esomeprazole	1929	10		
Pantoprazole	1093	6		
Rabeprazole	215	1		
Mixed use	2924	16		

Abbreviations: COPD=chronic obstructive pulmonary disorder; H2RA=histamine-2-antagonist; NSAID=non-steroidal anti-inflammatory drug; PPI=proton pump inhibitor.

North Jutland County, Denmark (1990–2003).

a Simultaneous prescription for any two of three antibiotics: amoxicillin, clarithromycin and metronidazole.

b ⩾1 year before censoring events.

c From public prescription and discharge registries.

d From the first two prescriptions registered.

**Table 2:** Adjusted rate ratios for gastric cancer for exclusive users of PPIs and H2RAs compared with non-users of both PPI and H2RA, and for PPI users compared with H2RA users

	**Lag time=1 Year^a^**		
	**Person-years**	**Gastric cancers**	**IRR (95% CI)^b^**
Unexposed	2 345 905	519	reference
			
*PPI vs Unexposed*			
2+ Prescriptions	51 854	24	1.2 (0.8–2.0)
*Years of follow-up*			
<1	13 316	10	2.3 (1.2–4.3)
1	10 391	3	0.8 (0.2–2.4)
2–4	19 363	4	0.5 (0.2–1.4)
5+	8783	7	2.3 (1.2–4.3)
*No. of prescriptions*			
2–4	30 588	8	0.8 (0.4–1.6)
5–14	13 851	9	1.6 (0.8–3.3)
15+	7415	7	2.1 (1.0–4.7)
			
*H2RA vs Unexposed*			
2+ Prescriptions	81 256	30	1.2 (0.8–1.8)
*Years of follow-up*			
<1	14 847	5	1.2 (0.5–2.8)
1	12 821	10	2.6 (1.4–4.9)
2–4	28 925	6	0.7 (0.3–1.5)
5+	24 663	9	1.1 (0.5–2.8)
*No. of prescriptions*			
2–4	43 060	9	0.8 (0.4–1.5)
5–14	24 213	13	1.7 (0.9–2.9)
15+	13 984	8	1.6 (0.8–3.2)
			
*PPI vs H2RA*			
2+ Prescriptions			1.3 (0.7–2.3)
*Years of follow-up*			
<1			2.4 (0.7–8.0)
1			0.7 (0.2–3.0)
2–4			0.8 (0.2–3.3)
5+			1.8 (0.6–5.0)
*No. of prescriptions*			
2–4			1.1 (0.4–3.2)
5–14			1.7 (0.7–4.5)
15+			1.4 (0.5–4.3)

Abbreviations: H2RA=histamine-2-antagonist; IRR=incidence rate ratio; PPI=proton pump inhibitor.

North Jutland County, Denmark (1990–2003).

a 1 year subtracted from diagnosis and censoring dates (except age and shift of treatment).

b Adjusted for: age, gender, calendar period, gastroscopy (⩾1 year before censoring events), use of NSAIDs and *H. pylori* eradication.
